# A common source for a trichinellosis outbreak reported in France and Serbia in 2017

**DOI:** 10.2807/1560-7917.ES.2020.25.24.1900527

**Published:** 2020-06-18

**Authors:** Régine Barruet, Alice Devez, Jean Dupouy-Camet, Gregory Karadjian, Dragana Plavsa, Georges Chydériotis, Isabelle Vallée, Ljiljana Sofronic-Milosavljevic, Hélène Yera

**Affiliations:** 1 Department of Internal Medicine, Hôpital André Grégoire, Montreuil, France; 2 Reference Laboratory for Human Trichinellosis, Hôpital Cochin, Hôpitaux Universitaires Paris centre, APHP, Faculty of Medicine, Université Paris Descartes, Paris, France; 3 National Reference Laboratory on Foodborne Parasites, ANSES, ENVA, UPEC, JRU BIPAR, Maisons-Alfort, France; 4 Institute of Public Health of Serbia “Dr Milan Jovanovic Batut”, Belgrade, Serbia; 5 Department of Immunology, Laboratoire Biomnis, Lyon, France; 6 National Reference Laboratory for Trichinellosis-NRLT, Institute for the Application of Nuclear Energy - INEP, University of Belgrade, Belgrade, Serbia

**Keywords:** trichinellosis, *Trichinella spiralis*, pork, France, Serbia

## Abstract

Trichinellosis is a rare parasitic zoonosis in the European Union. Meat from backyard pigs was the common source for a trichinellosis outbreak caused by *Trichinella spiralis*, which occurred in France and Serbia in the beginning of 2017. An epidemiological study was conducted in France and Serbia to determine the extent of the outbreak, to identify its source and to implement control measures. Three cases were exposed in Serbia and brought back to France pork delicatessen which they shared with relatives and friends. Around 47 individuals were exposed to the parasitised meat in France and Serbia and 20 cases of trichinellosis were reported (nine in France and 11 in Serbia). Nine of them were female. The diagnosis was delayed, in part because the parasitosis was not known by most physicians, which led to complications in the French cases such as facial paralysis and pulmonary embolism. Health alerts and survey networks are indispensable at a European level to control the disease.

## Background

Trichinellosis is a widespread helminthic zoonosis. Humans are accidental hosts contaminated by the ingestion of raw meat containing larvae. The disease can begin with intestinal disorders, followed by facial oedema, fever and finally myalgia and asthenia. It can lead to neurological and cardiac complications and can be lethal [1].

In 1992, the European Union (EU) Council Directive 92/45 required the examination of meat of wild boars, domestic pigs and horses for the presence of *Trichinella* spp. larvae before processing and marketing [2,3]. Since then, there has been a sharp decrease in human trichinellosis incidence rates in the EU and European Economic Area (EEA). However, trichinellosis is still a concern for Europe [4]. In 2017, 15 EU/EEA countries reported 224 cases of trichinellosis, of which 168 cases were confirmed [5]. Cases of trichinellosis imported from endemic countries are regularly reported in various European countries. These cases can be related to a local contamination while travelling or contamination after illegal importation of meat products by travellers. 

In France, between 1998 and 2016, 31 imported cases represented 39% of all cases reported to the National Reference Centre for Trichinellosis (NRCT) [6,7]. The war and social upheavals at the end of the 20th century in former Yugoslavia [8,9] led to a considerable local emergence of trichinellosis resulting in exported cases observed for instance in Germany [10], France [11] or the United Kingdom (UK) [12]. In the UK, an outbreak of nine cases was reported among Yugoslavian migrants in west London and in Hertfordshire after eating infected salami made from pork that was brought from Sombor in northern Serbia into the UK in November 1999 and given to four households. Since then, implementation of control methods in Serbia and Croatia has led to a sharp decrease of the disease but it still persists in some regions [13,14]. 

## Outbreak detection

A woman in her 40s was hospitalised on 7 February 2017 in the Department of Internal Medicine of a hospital in the suburbs of Paris (Case 1). Her relative, a man in his late 30s (Case 2), and her friend, a woman in her 60s (Case 3), were hospitalised on 21 and 24 February, respectively. Trichinellosis was suspected because of the combination of fever, facial oedema and eosinophilia. *Trichinella* serology was performed and was positive in the three patients, confirming the diagnosis. During the Christmas holidays, the patients had travelled to Serbia where they had eaten pork meat. They brought delicatessen back to France. The French health and veterinary agencies were alerted on 24 February and this alert was transmitted on 27 February to their Serbian counterparts. Analysis of the remaining meat in France allowed the identification of *T. spiralis*.

Here, we describe the epidemiological investigations undertaken in both countries to identify the extent and the source of the outbreak, implement appropriate control measures and our European cooperation.

## Methods

### Data sources

During this outbreak, the medical and epidemiological data were collected by staff of the hospital in France and Clinic X in Serbia. The collection of data was completed by the French reference laboratory for human trichinellosis (located at the Department of Parasitology, Cochin hospital, Paris and formerly, NRCT), the French health authorities (Santé Publique France), the Serbian National Reference Laboratory for Trichinellosis (NRLT INEP) and veterinary services in the Belgrade District.

### Case definition

Confirmed cases were defined as individuals with at least one of the following clinical symptoms: fever, facial oedema or myalgia, as well as elevated eosinophil counts (> 0.50 g/L), muscle enzymes creatine phosphokinase CPK (> 200 international units (IU)/L) and a positive serology in ELISA (Novalisa *Trichinella spiralis* IgG, Novatec, Orléans, France or INEP *Trichinella* ELISA test, Belgrade, Serbia) and or in immunoblot (LDBio Diagnostics, Lyon, France) [1].

### Outbreak investigation

An epidemiological study was conducted in order to determine the extent of the outbreak, to identify its source and to implement control measures.

Information on exposed people were obtained after interviews with Cases 1, 2 and 3. Moreover, people who the meat was shared with were asked if they shared it further.

In France, all people who had consumed the suspected meat were asked to consult the hospital treating the cases or their general practitioners, even if they were asymptomatic, in order to have a clinical examination and laboratory tests. In Serbia, it was recommended that exposed people should go for examination to Clinic X. Information was collected on age, sex, date of the first contaminated meal, date of onset of symptoms compatible with trichinellosis, symptoms, date and results of blood tests, treatment and time of hospitalisation.

### Laboratory tests

In France, an ELISA (Novalisa *Trichinella spiralis* IgG) was performed and confirmed by immunoblot (LDBio Diagnostics) [15]. Both reagents used excreted/secreted (E/S) antigens of *T. spiralis*. In Serbia, an ELISA (INEP *Trichinella* ELISA test) based on E/S antigens of *T. spiralis* was performed [16].

The remains of pork meat (ca 300 g) imported to France were analysed by the French National Reference Laboratory on food-borne parasites, Maisons-Alfort. Recommended methods were performed: artificial digestion to isolate the larvae, followed by species identification of the *Trichinella* larvae after DNA extraction and multiplex PCR [3,17,18]. Viability and infectivity of the larvae were evaluated by mouse bioassay, consisting of the inoculation of a mouse with the isolated larvae and analysis of the presence of larvae in muscle five weeks after infection [19].

The Serbian veterinary authorities identified owners of the implicated pigs and seized the remaining meat products. Samples of smoked and fresh meat of one implicated pig (200 kg of body weight at slaughter) were analysed by the NRLT INEP with the recommended methods.

### Statistical analysis

The attack rate and the frequency of trichinellosis characteristics in the French cases and the Serbian cases were compared using Fisher’s exact test. The mean days in hospital in the two groups of cases were compared using Student’s t-test. A probability of 0.05 or less was considered to be significant in the tests.

### Ethical statement

Approval for communicable disease outbreak investigations was given by the French and Serbian health agencies in the public interest. The French reference laboratory for human trichinellosis, the French health authorities, the French National Reference Laboratory on food-borne parasites, the Serbian National Reference Laboratory for Trichinellosis and veterinary services in the Belgrade District had the agreements of their respective national ministry to work on and to investigate *Trichinella* outbreaks without any registration number. Informed consent of patients was obtained before investigation. National standards for the care and use of animals were followed and the animal experiments were approved by an ethical review committee (C2EA-16 Comité d’éthique ComEth ANSES/ENVA/UPEC, under the approval number: saisine 12-0048, ComEth 13/11/12-4).

## Results

### Chronology of the whole event

The suspected meat came from three backyard pigs bred on a family-owned small farm in a village near Belgrade. The meat from one pig was prepared as canned food (sausages and dried meat), the second was cooked and the third prepared as smoked food. Cases 1, 2 and 3 had travelled to Serbia during the Christmas holidays. They had eaten pork meat from the farm on 31 December 2016 and on 1 and 7 January 2017. They brought back to France meat from the first and second pigs. In a city in the suburbs of Paris, they shared these delicatessen with 19 individuals (friends, relatives and colleagues) between 9 and 20 January. In Serbia, 25 additional individuals (relatives and friends) consumed meat from three pigs during one or more meals between 31 December and 15 January.

### Clinical and laboratory findings

#### French investigation

The index case was severe with abdominal pain, diarrhoea, prolonged fever (4 weeks), myalgia, facial oedema, productive cough, rhinorrhoeas and major asthenia (Table 1). Her symptoms began on 27 January (11 days before consulting a doctor) (Figure). This case was complicated by pulmonary embolism. Of note, although this patient had a known deficiency in protein S and travelled back from Serbia by car without any anti-thrombotic prevention, no inferior limb phlebitis could be detected. At blood examination, she had high blood eosinophil counts and elevated muscle enzymes. On 10 February, serology for trichinellosis was equivocal in an ELISA but positive in immunoblot. Case 2 had similar clinical signs without complications, but did not consult a doctor until 24 February. Case 3 had fever for just 1 day but diffuse myalgia and prolonged deep asthenia. She consulted a doctor on 21 February. Cases 2 and 3 also had elevated levels of eosinophils and muscle enzymes and positive serology for trichinellosis (Table 1).

**Table 1:** Clinical and laboratory data of confirmed trichinellosis cases observed in France (Cases 1 to 9) and in Serbia (Cases 10 to 20), January–February 2017 (n = 20)

Case	Sex	Age group (years)	Meat consumption	Symptoms	Complications	Days in hospital	Eosinophils (g/L)/CPK (IU/L)^a^	ELISA result	IB result
1	F	40–49	Sausages, dried and smoked pork	D, Fe (4 weeks), My, O	Pulmonary embolism	14	8.35/685	+ / −	+
2	M	30–39	Sausages, dried and smoked pork	D, Fe (2 weeks), My, O	None	7	2.15/3,081	+	+
3	F	60–69	Sausages, dried and smoked pork	Fe (1 day), My	None	9	1.38/382	−	+
4	F	30–39	Sausages, dried pork	Fe (2 weeks), My, O, lO	None	11	3.52/633	+	+
5	M	60–69	Sausages, dried pork	Fe, My	Facial paralysis	8	0.20/118	−	+
6	M	40–49	Sausages, dried pork	D, Fe (3 days), My	None	5	7.2/293	+	+
7	F	40–49	Sausages, dried pork	Fe, My, O, lO	None	6	3.53/206	+	+
8	M	50–59	Sausages, dried pork	Fe (> 1 week), My, pO	None	8	5.80/462	+	+
9	M	10–19	Sausages, dried pork	D, Fe (1 day)	None	3	3.04/1,433	+	+
10	F	50–59	Sausages, dried and smoked pork	Fe, D, My, O	None	0	3.37/nda^b^	+	nd
11	F	10–19	Sausages, dried and smoked pork	Fe, D, My, O	None	0	4.55/nda^b^	+	nd
12	F	10–19	Sausages, dried and smoked pork	Fe, D, My, pO	None	0	2.12/nda^b^	+	nd
13	M	40–49	Smoked pork	Fe, D, My, O, lO, pO	None	0	Eo+^c^/nda^b^	+	nd
14	M	10–19	Smoked pork	Fe, D, My, O, lO, pO	None	0	Eo+/nda^b^	+	nd
15	M	<10	Smoked pork	Fe, D, My, O, lO, pO	None	0	Eo+/nda^b^	+	nd
16	M	10–19	Smoked pork	Fe, D, My, pO	None	0	Eo+/nda^b^	+	nd
17	F	10–19	Smoked pork	Fe, D, My, pO	None	0	Eo+/nda^b^	+	nd
18	M	10–19	Smoked pork	Fe, My, pO	None	0	Eo+/nda^b^	+	nd
19	F	30–39	Smoked pork	Fe, My, pO	None	0	Eo+/nda^b^	+	nd
20	M	40–49	Smoked pork	Fe, My, pO	None	0	Eo+/nda^b^	+	nd

CPK: creatine phosphokinase; D: diarrhoea; ELISA: enzyme-linked immunosorbent assay; F: female; Fe: fever; IB: immunoblot; IU: international units; lO: limb oedema; M: male; My: myalgia; nd: not done; nda: no data available; O: facial oedema; pO: periorbital oedema.

^a^ Normal ranges: eosinophils 0.04–0.40 g/L, CPK 10–200 IU/L.

^b^ nda: no data available/lack of data for ambulatory treated patients, records were kept only for 1 year because of the clinic’s internal procedure.

^c^ Eo+ : Eosinophilia detected and noted in reports but no exact data were available 2 years after examination of patients.

**Figure:**
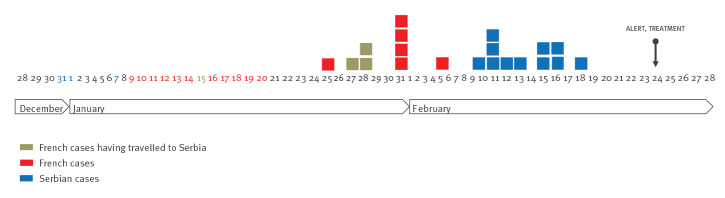
Epidemic curve of the trichinellosis outbreak, France and Serbia, December 2016–February 2017 (n = 20)

Dates of contaminated meals are represented in colour: blue: meals in Serbia, red: meals in France, olive: meal in both countries. Cases are shown by date of symptom onset.

Among the 19 other people exposed in France, two were lost to follow-up, seven were asymptomatic and 10 presented clinical signs compatible with trichinellosis from mid-January to the end of February. Trichinellosis was excluded in four symptomatic persons because of negative serology, normal blood eosinophils counts and muscle enzyme levels determined in sera collected 50–60 days after eating contaminated meat. The six additional cases identified by this survey (Cases 4 to 9) had a confirmed infection, as five had elevated levels of eosinophils and muscle enzymes and all had positive serology for trichinellosis (Table 1). Case 5 was positive in serology but had normal levels of eosinophils and muscle enzymes. He received high doses of corticosteroids for 8 days for facial paralysis. This patient had had a previous facial paralysis episode 2 years before and the magnetic resonance imaging (MRI) prescribed during the present episode did not show any abnormalities. Myocarditis was suspected in Case 6 (high levels of troponin) and Case 7 (bundle branch block and limb oedema) but excluded after thorough cardiological investigations. Case 8 had periorbital oedema that led the patient to consult an ophthalmologist at the end of January but the diagnosis of trichinellosis was not made on that occasion. Case 9 had limited clinical signs, denied having eaten parasitised pork but had high eosinophil counts and positive serology.

The attack rate in the French patients was 45% (9/20). The incubation time was between 16 and 28 days: 27 and 28 days in Cases 1, 2 and 3 and 16–23 days in the other cases. Sera were collected between 41 and 62 days after the first contaminated meal. ELISA was positive in seven of the nine cases, while immunoblot was positive in all cases. All cases were hospitalised at the same hospital for 3–14 days and were treated for 15 days with albendazole (15 mg/kg/day) and prednisolone (1 mg/kg/day for a few days and decreasing doses afterwards). Treatments were prolonged for 1 more week for Cases 1 and 8 because of persisting symptoms.

The rest of the pork meat was positive for the presence of *Trichinella* larvae, identified as *T. spiralis*. Sausages and dried meat contained 51 and 62 larvae per gram, respectively. The mouse bioassay was negative, suggesting that the larvae were not infectious at the time when the meat was analysed, i.e. 2 months after the first meat consumption.

#### Serbian investigation

As a result of the outbreak detection in France and the alert transmitted to the Serbian authorities on 27 February, the local veterinary inspector and epidemiologist advised the family of Cases 1 and 2 and family guests to consult at Clinic X in Belgrade.

Among 25 people exposed in Serbia, 11 had trichinellosis (Cases 10-20). They had symptoms compatible with trichinellosis, elevated levels of eosinophils and positive serology for trichinellosis (Table 1). ELISA was positive in all cases from 44 to 61 days after meat consumption.

The cases were members of the family of Cases 1 and 2 (Cases 10–12) and eight family guests (Cases 13–20). During examinations at the clinic in Serbia at the beginning of March, the general health parameters in all patients were satisfactory and the symptoms of *Trichinella* infection had passed. The symptoms they described began between 10 and 18 February (Figure), were mild and reminded them of the symptoms of influenza (Table 1). A total of 25 individuals had been exposed to the contaminated meat and the attack rate was 44% (11/25). The incubation time was estimated between 34 and 42 days. All cases were treated with albendazole and corticoids in ambulatory care but they were not hospitalised.

The French people having travelled in Serbia (Cases 1, 2 and 3) had not been included in the description of Serbian cases.

The presence of *Trichinella* larvae was detected in all samples of smoked and fresh meat from the third pig. The larval burden ranged from 21 to 61 larvae per gram. *T. spiralis* larvae were also identified by multiplex PCR. According to the farmer, the meat from the present outbreak was sent after slaughter for analysis to a veterinarian who informed the family by telephone that the meat was not infected, but did not send an official written confirmation. Although the analysis was done by trichinelloscopy, a non-recommended method, it would have been sensitive enough to detect the high larval burden of the incriminated meat ranging from 21 to 62 larvae per gram.

#### Comparison of French and Serbian cases

Of the 20 cases, nine were female. When comparing the characteristics of trichinellosis in both countries, we identified a few differences. The French cases had higher mean and median ages (17–61 years vs 8–56 years). They had less periorbital oedema and more of them were hospitalised (p < 0.010) (Table 2).

**Table 2:** Characteristics of trichinellosis according to the countries, France and Serbia, January–February 2017 (n = 20)

Characteristics	Total cases (n = 20)	French cases (n = 9)	Serbian cases (n = 11)	p value^a^
Mean age (years)	34	44.33	25.55	Not applicable
Median age (years)	37	46	17	Not applicable
Female	9	4	5	1.00
Clinical data				
Fever	20	9	11	1.00
Myalgia	19	8	11	0.45
Facial oedema	9	4	5	1.00
Periorbital oedema	10	1	9	< 0.01
Diarrhoea	12	4	8	0.36
Limb oedema	5	2	3	1.00
Complications	2	2	0	0.19
Hospitalisation	9	9	0	< 0.01
Mean number of days in hospital	3.55	7.89	0	< 0.01
Laboratory data				
Eosinophilia	19	8	11	0.45
Positive serology	20	9	11	1.00

^a^ French cases vs Serbian cases.

The attack rates were similar in both countries (44 and 45%) (p = 1.000). The young cases (< 30 years-old) were mild in both countries (Table 1).

### Outbreak control measures

As soon as the outbreak was detected, the families were asked to stop the consumption of the contaminated meat and were informed about the risks of eating contaminated undercooked meat. The remaining meat were seized and destroyed. All veterinarians in the Belgrade district were warned to apply the recommended methods of detecting *Trichinella* larvae in meat.

The information was quickly exchanged between the French and Serbian *Trichinella* specialists and the national authorities. This allowed us to control the outbreak.

## Discussion

This outbreak confirms that travel in endemic regions is a classical driver for acquiring trichinellosis [6]. Travellers should be informed of the risks of eating raw meat products bought outside of the official market (uninspected) in countries with high prevalence of trichinellosis (south-east Balkan countries: Bulgaria, Romania, Serbia). The outbreak described here was one of two that took place in Serbia in 2017. The source of contamination was back-yard pigs bred in a private farm in the suburbs of Belgrade. The probable absence of meat inspection and the low awareness of trichinellosis risk led to the consumption of the infected meat. The International Commission on Trichinellosis and the European Union Reference Laboratory for Parasites recommend the use of the more sensitive artificial digestion method [3]. Moreover, in France as in Serbia, parasitological inspection of meat destined for human consumption outside the family is obligatory [2].

The incidence of trichinellosis in France is low, with only 68 cases reported from 2001 to 2016. Among these 68 cases, 38 were related to the consumption of infected meat abroad or to illegally imported meat [7]. Serbia on the other hand represents a high-risk country for trichinellosis. During the period 2001 to 2016, there were 2,897 cases of human trichinellosis (including three deaths in 2005) [20,21]. The number of infected people varied over the years, with the maximum number reported in 2002 (577 cases) and the minimum number reported in 2012 (46 cases). The main source of trichinellosis (in more than 90% of cases) was meat or meat products containing *T. spiralis* and prepared from domestic swine raised on small private farms and not subjected to parasitological investigation (avoided by farmers). This kind of homemade meat products intended for personal usage is often distributed among relatives and friends in Serbia and abroad. Other sources of trichinellosis in Serbia, include wild boar (ca 5% of cases) and horse meat (less than 2% of cases) [13].

In this outbreak, the diagnosis of trichinellosis was delayed, as it was done on 24 February while it could have been done at the end of January. Various reasons could explain this delay. Firstly, the three first cases sought medical advice 11, 24 and 27 days after the beginning of symptoms. Secondly, patients consulted physicians not familiar with this rare disease in France. Indeed, when Case 5 consulted his general practitioner for facial paralysis, he received only a high dose of corticoids modifying the immune response and leading to normal blood eosinophil counts and muscle enzymes and delaying the appearance of specific antibodies. When Case 8 consulted an ophthalmologist for periorbital oedema, trichinellosis was not suspected. Thirdly, antibodies usually appear a few days after the onset of the acute phase of the disease, leading to the frequent observation of a negative serology at the beginning of the symptoms. A second assay is recommended to confirm a seroconversion which has a high diagnosis value [1]. Seroconversion usually occurs between 2 and 5 weeks after infection [1]. In case of high suspicion, immunoblot should be performed systematically (but not as a confirmatory method). Indeed, it can be used earlier and is more sensitive than ELISA for the detection of anti-*Trichinella* IgG in the acute phase [15,22]. Here, we also observed that some cases (Cases 3 and 5) had a positive serology with immunoblot but not in ELISA. Fourthly, this outbreak occurred during the usual period for influenza which can cause symptoms similar to those of trichinellosis. Finally, some patients of Serbian origin were not fluent in French and this could have explained some misunderstanding of the symptoms.

While the cases in Serbia were moderate, the cases in France were severe, as all patients were hospitalised but no major complications were observed. The pulmonary embolism in Case 1 could have been triggered by a previously known protein S deficiency. Although high levels of troponin have been reported as signs of myocarditis during trichinellosis [23], myocarditis was in the present outbreak excluded in Case 6 who had elevated levels. 

The difference in the severity of the disease between the two countries could be explained by the type of meat that was consumed. The cases in Serbia consumed mainly smoked pork, while dried pork was consumed in France. Traditionally, Serbian homemade smoked meat is prepared by using first a dry-salting process (10% salt by weight of meat, 7–14 days), followed by a wet-salting process (0.5% salt/L of water, 7 days) and then followed by cold smoking (3–4 weeks). In this outbreak, smoked meat was prepared in less than 20 days (salting and smoking were shortened), the sausages were exposed only to quick cold smoking and dried meat was prepared by quick dry salting. The time of drying may not have been long enough to inactivate the larvae, while smoking may have inactivated more larvae by inducing a quicker dehydration. The survival of *Trichinella* in meat depends on the remaining moisture content [24]. However, we could not confirm this hypothesis. Nevertheless, the longer incubation time of trichinellosis in Serbian vs French cases suggested that the infectious dose was lower in the smoked pork. In any case, this outbreak showed that smoking and drying are not sufficient to inactivate the parasite, although the mouse bioassay was negative. 

Another hypothesis could be that the majority of the French cases were older than the Serbian cases (8/9 vs 4/11 were older than 30 years) and consequently, they had more risk of complications [1]. A final hypothesis could be that the Serbian cases had had previous contacts with *Trichinella* parasites and developed an immunity, leading to a longer incubation phase and a milder symptomatology in case of reinfections. Such an immunity has been observed in Inuit populations having frequent contact with infected meat [25]. We recently reported three cases of trichinellosis related to consumption of polar bear meat in East Greenland [26]. One of the three cases had no clinical signs but only elevated eosinophils. An immunoblot assay performed several months earlier showed that this frequent traveller to Greenland already had *Trichinella* antibodies. However, the difference in hospitalisation rates between the two countries we report here could also be attributed to a different perception of the illness or the fact that the French cases were complicated.

Here, we could not determine the dose of parasites to which the patients were exposed since the worm burden varies between different pieces of meat and we did not collect information on the precise amount of meat consumed by the patients repeatedly over several days. 

An early diagnosis leading to early treatment with albendazole and corticosteroids prevents the occurrence of complications [1]. Fortunately, the international network of *Trichinella* specialists together with the national authorities facilitated the investigation of this outbreak involving two European countries.

## References

[1] Dupouy-Camet J, Bruschi F. Management and diagnosis of human trichinellosis. In: FAO/WHO/OIE guidelines for the surveillance, management, prevention and control of trichinellosis. Dupouy-Camet J. Murrell K.D. (editors). Paris: World Organisation for Animal Health; 2007. pp 37-68.

[2] Council of the European Communities. Council Directive 92/45/EEC of 16 June 1992, on public health and animal health problems relating to the killing of wild game and the placing on the market of wild-game meat. Official Journal of the European Union. Luxembourg: Publications Office of the European Union. 14. 9. 92:L 268. Available from: http://eur-lex.europa.eu/legal-content/EN/TXT/?qid=1426636760883&uri=CELEX:31992L0045

[3] European Commission. Commission implementing regulation (EU) 2015/1375 of 10 August 2015 laying down specific rules on official controls for Trichinella in meat. Official Journal of the European Union. Luxembourg: Publications Office of the European Union. 11.8.2015:L 212. Available from: http://eur-lex.europa.eu/legal-content/EN/TXT/?qid=1448152892014&uri=CELEX:32015R1375

[4] Dupouy-Camet J Trichinellosis: still a concern for Europe. Euro Surveill. 2006;11(1):590. 10.2807/esm.11.01.00590-en 16484733

[5] European Centre for Disease Prevention and Control (ECDC). Trichinellosis - Annual epidemiological report for 2017. Stockholm: ECDC; 2019. Available from: https://ecdc.europa.eu/en/publications-data/trichinellosis-annual-epidemiological-report-2017

[6] Dupouy-Camet J Travels and tourism are drivers for trichinellosis. Parasitol United J. 2014;7(2):86-92. 10.4103/1687-7942.149555

[7] French Reference Laboratory on Human Trichinellosis (FRLT). Cases of trichinellosis published or notified in France between 1975 and 2016. Paris FRLT; 2016. Available from: https://cnrdestrichinella.monsite-orange.fr/page3/index.html

[8] Cuperlovic K Djordjevic M Pavlovic S Sofronic-Milosavljevic L Present status of trichinellosis in Yugoslavia: Serbia. Parasite. 2001;8(2) Suppl;S95-7. 10.1051/parasite/200108s2095 11484397

[9] Djordjevic M Bacic M Petricevic M Cuperlovic K Malakauskas A Kapel CM Social, political, and economic factors responsible for the reemergence of trichinellosis in Serbia: a case study. J Parasitol. 2003;89(2):226-31. 10.1645/0022-3395(2003)089[0226:SPAEFR]2.0.CO;2 12760633

[10] Nothdurft HD Brommer M Eichenlaub D Löscher T [A small outbreak of trichinosis caused by imported smoked ham]. Dtsch Med Wochenschr. 1995;120(6):173-6. German. 10.1055/s-2008-1055330 7851288

[11] Lefort A Lortholary O Dupouy-Camet J Rousset JJ Guillevin L Imported trichinellosis from former Yugoslavia. Clin Microbiol Infect. 1997;3(4):506-7. 10.1111/j.1469-0691.1997.tb00295.x 11864169

[12] Milne LM Bhagani S Bannister BA Laitner SM Moore P Eza D Trichinellosis acquired in the United Kingdom. Epidemiol Infect. 2001;127(2):359-63. 10.1017/S0950268801005994 11693515 PMC2869757

[13] Sofronic-Milosavljevic Lj Djordjevic M Plavsic B Grgic B Trichinella infection in Serbia in the first decade of the twenty-first century. Vet Parasitol. 2013;194(2-4):145-9. 10.1016/j.vetpar.2013.01.042 23462255

[14] European Food Safety Authority (EFSA) The European Union summary report on trends and sources of zoonoses, zoonotic agents and food-borne outbreaks in 2017. EFSA J. 2018;16:5500. 10.2903/j.efsa.2018.5500 PMC7009540 32625785

[15] Yera H Andiva S Perret C Limonne D Boireau P Dupouy-Camet J Development and evaluation of a Western blot kit for diagnosis of human trichinellosis. Clin Diagn Lab Immunol. 2003;10(5):793-6. 10.1128/CDLI.10.5.793-796.2003 12965906 PMC193884

[16] Gnjatovic M Gruden-Movsesijan A Miladinovic-Tasic N Ilic N Vasilev S Cvetkovic J A competitive enzyme-linked immunosorbent assay for rapid detection of antibodies against Trichinella spiralis and T. britovi - one test for humans and swine. J Helminthol. 2019;93(1):33-41. 10.1017/S0022149X17001092 29168448

[17] Zarlenga DS Chute MB Martin A Kapel CM A multiplex PCR for unequivocal differentiation of all encapsulated and non-encapsulated genotypes of Trichinella. Int J Parasitol. 1999;29(11):1859-67. 10.1016/S0020-7519(99)00107-1 10616932

[18] European Union Reference Laboratory for Parasites, Istituto Superiore di Sanità (ISS). Identification of Trichinella muscle stage larvae at the species level by multiplex PCR. Rome: ISS; 2013. Available from: http://www.iss.it/binary/crlp/cont/MI_02_WEB_SITE.pdf

[19] Kapel CM Measures L Møller LN Forbes L Gajadhar A Experimental Trichinella infection in seals. Int J Parasitol. 2003;33(13):1463-70. 10.1016/S0020-7519(03)00202-9 14572509

[20] Sofronic-Milosavljevic Lj, Vasilev S, Cvetkovic J, Mitic I, Plavsa D, Andric A, et al. Trichinella infection in Serbia in 2016. 12th Workshop of National Reference Laboratories for Parasites. Rome: Istituto Superiore di Sanità; 18-19 May 2017.

[21] Pavic S Andric A Sofronic-Milosavljevic LJ Gnjatovic M Mitić I Vasilev S Trichinella britovi outbreak: Epidemiological, clinical, and biological features. Med Mal Infect. 2019;S0399-077X(19)31060-1. 10.1016/j.medmal.2019.10.008 31732242

[22] Gari-Toussaint M Tieulié N Baldin J Dupouy-Camet J Delaunay P Fuzibet JG Human trichinellosis due to Trichinella britovi in southern France after consumption of frozen wild boar meat. Euro Surveill. 2005;10(6):117-8. 10.2807/esm.10.06.00550-en 16077211

[23] Lachkar S Abboud P Gargala G Etienne M Gauliard E Tron C [Troponin dosage in a patient with asymptomatic myocarditis due to trichinellosis]. Rev Med Interne. 2008;29(3):246-8. French. 10.1016/j.revmed.2007.07.011 17980464

[24] Gamble HR, Boireau P, Kockler K, Kapel CMO. Prevention of Trichinella infection in the domestic pig. In: FAO/WHO/OIE guidelines for the surveillance, management, prevention and control of trichinellosis. Dupouy-Camet J. Murrell K.D. (editors). Paris: World Organisation for Animal Health; 2007. pp 99-108.

[25] Viallet J MacLean JD Goresky CA Staudt M Routhier G Law C Arctic trichinosis presenting as prolonged diarrhea. Gastroenterology. 1986;91(4):938-46. 10.1016/0016-5085(86)90698-0 3743971

[26] Dupouy-Camet J Yera H Dahane N Bouthry E Kapel CMO A cluster of three cases of trichinellosis linked to bear meat consumption in the Arctic. J Travel Med. 2016;23(5):1-3. 10.1093/jtm/taw037 27296583

